# The Oral Cavity and Age: A Site of Chronic Inflammation?

**DOI:** 10.1371/journal.pone.0001351

**Published:** 2007-12-26

**Authors:** Magnus Bäck, Hanna Hlawaty, Carlos Labat, Jean-Baptiste Michel, Charles Brink

**Affiliations:** 1 INSERM U698, University of Paris 7, Bichat Hospital, Paris, France; 2 University of Paris 13, Institut Galilée UP13, Villetauneuse, France; 3 INSERM U684, Nancy, France; 4 Henri Poincare University, Nancy, France; University of Minnesota, United States of America

## Abstract

**Background:**

Aging may be accompanied by a low grade chronic up-regulation of inflammatory mediators. A variety of endogenous locally released mediators as well as inflammatory cells have been reported in the human oral cavity. The aim of this investigation was to determine the presence of different classes of inflammatory mediators in human saliva and correlate the levels with age.

**Methodology and Principal Findings:**

Unstimulated whole buccal salivary samples were obtained in the morning from 94 healthy volunteers within 30 minutes after waking. None of the participants had taken aspirin in the week prior to the saliva collection. Lysozyme activity, eicosanoid levels (prostaglandin E_2_ and leukotriene B_4_) and MMP-9 activity were measured. The antimicrobial activity (lysozyme activity) was not correlated with age whereas PGE_2_ levels were markedly correlated with age (r = 0.29; P<0.05; n = 56). Saliva from healthy subjects (≤40 years) compared with data derived from older volunteers (>40 years) demonstrated a significant increase in the mean values for PGE_2_ and MMP-9 activity with age. In addition, significant correlations were observed between LTB_4_ and PGE_2_ (r = 0.28; P<0.05; n = 56) and between LTB_4_ levels and MMP-9 activity in smokers (r = 0.78; P<0.001; n = 15).

**Conclusions/Significance:**

The presence of significant levels and activity of inflammatory mediators in saliva suggests that the oral cavity of healthy subjects may be in a constant low state of inflammation associated with age.

## Introduction

The cardinal sign of inflammation is the presence of endogenous locally released mediators at the site of a perturbation. This initial event is followed by a more chronic condition characterized by a recruitment and accumulation of inflammatory cells associated with local proteolytic and oxidative activities at the site of insult. There is sufficient information suggesting that aging is accompanied by a low grade chronic up-regulation of inflammatory mediators [1]. Such underlying modifications may be responsible for, or contribute to, the evolution of various age-dependent disease conditions, such as, atherosclerosis.

In the oral cavity of normal healthy subjects, the presence of a spectrum of bacterial populations [2] and a number of leukocytes, especially polymorphonuclear leukocytes (PMNs) [3], [4] have been reported. In addition to these observations, human saliva contains several factors which can be classified and profiled according to their principal actions [5]. Lysozyme is known to have antimicrobial activities, whereas eicosanoids (prostaglandins and leukotrienes) are thought to have pro-inflammatory actions and matrix metalloproteinases (MMPs) [6] exhibit proteolytic effects at the site of inflammation.

In spite of the fact that these mediators have such remarkably diverse activities, there have been few attempts to describe correlations among them. Generally, most studies have simply targeted the role of a particular mediator as a marker of a disease condition [7]. However, the profiles of inflammatory cells and mediators are known to be altered during the progression of a disease and may explain the controversy that frequently arises among studies concerning similar patient cohorts [1]. Although the local inflammatory environment of the oral cavity has been reported to be markedly exacerbated in diseased subjects [6], [8]–[11], the phenomenon, albeit less intense, may also be present in healthy subjects of different ages. While there is considerable work published on disease in the oral cavity, there is little information available to elucidate the low chronic inflammatory conditions that may exist in the oral cavity in normal subjects. Indeed, most studies in patients have dealt only with the effects of the mediators of inflammation on acute phenomenon [2], [6] and few investigations have centered on the side effects of inflammatory mediators when the onset is slow but persistent over a prolonged period [7], [8], [11]. This study was undertaken to measure various mediators, known to be intimately linked to the inflammatory process, in saliva of healthy subjects at different ages. The principal aim was to establish the correlations which may exist among the different classes of mediators and aging with the hypothesis that the oral cavity may be in a state of low chronic inflammation.

## Results

There was considerable variation in the levels of lysozyme and MMP-9 activities as well as the levels of arachidonic acid metabolites detected in the human salivary samples under basal conditions (Table 1). However, the significantly higher levels of LTB_4_ compared with PGE_2_ (Table 1) was consistent in all subgroups studied (Table 2–4).

**Table 1:** Mediator levels and activities in salivary samples from all subjects.

Women Smokers	52%		
	22%		
	N	mean±sem	(range)
**Age** (years)	94	36±1	(14–76)
**Lysozyme activity** (µg/ml)	94	108±12	(1–821)
**PGE_2 _**(pg/ml)	56	242±31	(47–1126)
**LTB_4 _**(pg/ml)	60	1223±170	(105–6686)
**pro-MMP-9** (AU)	27	12.02±2.49	(0.01–51.65)
**MMP-9** (AU)	27	5.39±1.14	(0.00–20.01)

n indicates number of subjects studied.

**Table 2:** Mediator levels and activities in salivary samples from young (≤40 years) and old (>40 years) subjects.

Women Smokers	≤40 years			>40 years		
	48%			59%		
	21%			25%		
	n	means±sem	(range)	n	means±sem	(range)
**Age** (years)	62	27±1	(14–40)	32	54±1	(42–76)
**Lysozyme activity** (µg/ml)	62	119±17	(1–821)	32	86±14	(5–322)
**PGE_2 _**(pg/ml)	30	163±17	(59–381)	26	334±59*	(47–1126)
**LTB_4 _**(pg/ml)	33	1003±193	(105–4897)	27	1513±286	(217–6686)
**pro-MMP-9** (AU)	15	6.76±1.63	(0.01–20.13)	12	18.59±4.66*	(0.03–51.65)
**MMP-9 **(AU)	15	2.80±0.91	(0.01–13.52)	12	8.63±1.98*	(0.00–20.01)

n indicates number of subjects studied and

* indicates values significantly different from young subjects (P<0.05).

**Table 3 pone-0001351-t003:** Mediator levels and activities in salivary samples from non-smokers and smokers.

Women	Non-smokers			Smokers		
	51%			57%		
	n	means±sem	(range)	n	means±sem	(range)
**Age** (years)	73	36±2	(14–76)	21	38±3	(21–61)
**Lysozyme activity** (µg/ml)	73	114±14	(1–821)	21	86±21	(14–322)
**PGE_2 _**(pg/ml)	36	280±45	(47–1126)	20	175±27	(59–521)
**LTB_4 _**(pg/ml)	39	1237±228	(100–6686)	21	1224±228	(200–4718)
**pro-MMP-9** (AU)	12	10.37±2.61	(0.01–28.76)	15	13.34±4.02	(0.09–51.65)
**MMP-9** (AU)	12	5.01±1.52	(0.00–18.32)	15	5.69±1.70	(0.03–20.01)

n indicates number of subjects studied.

**Table 4:** Mediator levels and activities in salivary samples from men and women.

Smokers	Men			Women		
	20%			24%		
	n	means±sem	(range)	n	means±sem	(range)
**Age** (years)	45	35±2	(14–67)	49	37±2	(14–76)
**Lysozyme activity** (µg/ml)	45	83±11	(4–310)	49	130±21	(1–821)
**PGE_2 _**(pg/ml)	27	210±30	(51 -602)	29	273±53	(47–1126)
**LTB_4 _**(pg/ml)	28	1150±220	(201–4718)	32	1304±254	(100–6686)
**pro-MMP-9** (AU)	11	9.39±2.91	(0.03–28.00)	16	13.83±3.71	(0.01–51.65)
**MMP-9** (AU)	11	4.36±1.57	(0.00–16.53)	16	6.10±1.62	(0.01–20.01)

n indicates number of subjects studied.

**Figure 1:**
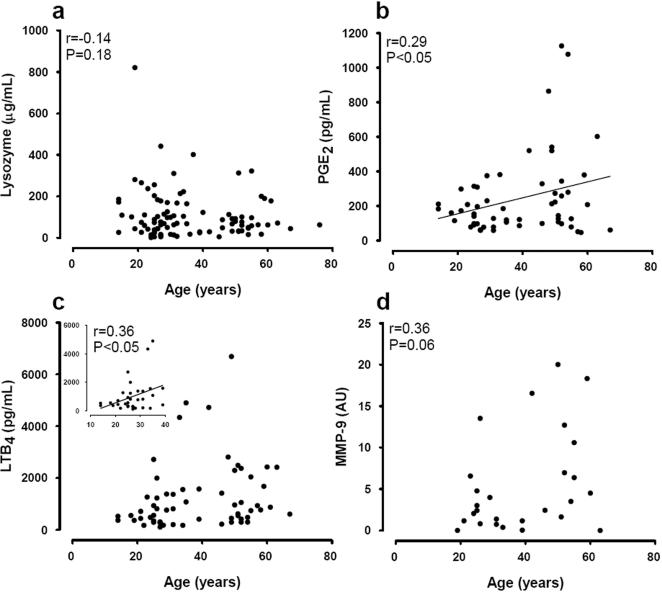
Relationship between age and mediators of inflammation in saliva from healthy subjects. Panel a: lysozyme activity, b: PGE_2_, c: LTB_4_ (with inset showing ≤40 years) and d: MMP-9 activity. Diagonal lines are the regression correlations when shown to be significant.

The lysozyme activity measured in human saliva was not correlated with age (Fig 1a; n = 94). In contrast, the data presented in Figure 1 indicate a significant correlation with age for PGE_2 _(Fig 1b; n = 56) with about 2-fold higher salivary levels of PGE_2_ in the subjects older than 40 years of age compared with those below 40 years (Table 2).

In contrast, LTB_4_ was not significantly correlated with age (Fig 1c; n = 60) and the salivary levels of LTB_4_ were not significantly different between older and younger subjects (Table 2). However, in the younger subjects, LTB_4_ was significantly correlated with age, although this may be due to the presence of outliers (Fig 1c inset; n = 33). Furthermore, a significant (approximately 3-fold) increase in MMP-9 activity was demonstrated in saliva derived from older (>40 years old) compared with younger (<40 years) subjects (Table 2).

**Figure 2:**
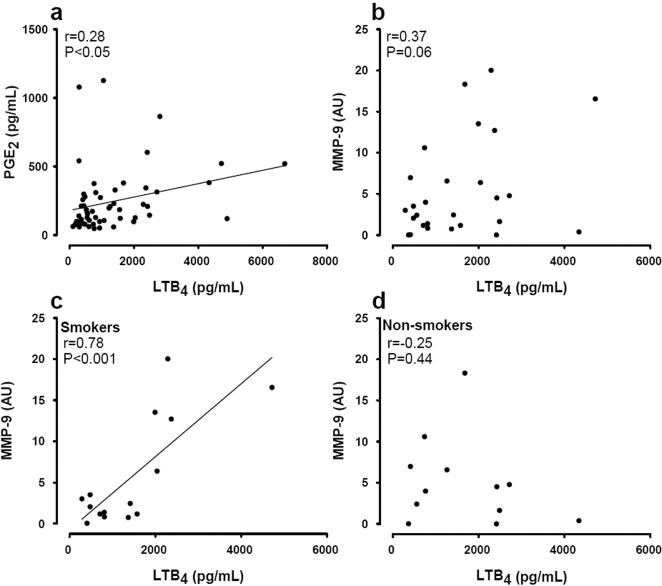
Relationship between different mediators of inflammation in saliva from healthy subjects. Panel a: PGE_2_ vs. LTB_4_, b: MMP-9 activity vs. LTB_4_, c MMP-9 activity vs. LTB_4_ in smokers, d: c MMP-9 vs. LTB_4_ in non-smokers. Diagonal lines are the regression correlations when shown to be significant.

The results presented in Figure 2 demonstrated significant correlations between the levels of the eicosanoids PGE_2 _and LTB_4_ detected in the human salivary samples (Fig 2a; n = 56). Although none of the mediators were significantly altered in saliva from smokers compared with non-smokers, the correlation between LTB_4_ levels and MMP-9 activity was revealed significant in the smoking group (Fig 2c, n = 15) but not in non-smokers. There were no significant correlations between the above mentioned mediators in relation to lysozyme (data not shown).

There was no significant modification in any of the mean values when the data were adjusted for either sex or smoking (Table 3 and 4). In addition, gelatin zymography demonstrated the presence of gelatinolytic activities in all saliva samples at apparent molecular weights of 92 kD and 80 kD corresponding to latent and active MMP-9, respectively. EDTA but not Trasylol®, inhibited these gelatinolytic activities (data not shown) supporting the observation that these effects were related to the MMP profiles.

## Discussion

The present study, demonstrating significant correlations between age and inflammatory mediators of the eicosanoid and MMP pathways in whole buccal saliva from healthy volunteers, points to a marked modification of the inflammatory state of the oral cavity associated with age. Furthermore, the data suggest that this age-related inflammatory response was independent of the innate oral antimicrobial activity as monitored by lysozyme activity.

Whereas the majority of previous investigations involving measurements of lysozyme activity in human saliva have dealt with either the cellular origin [12], [13] or the activities monitored in a variety of disease conditions in the oral cavity [2], [7], [8], [14]–[16], there is little conclusive information on the lysozyme activity in saliva from normal subjects and age. The present data from healthy volunteers demonstrated that the lysozyme activity was not correlated with age. In contrast, PGE_2_ levels and MMP-9 activity were increased with age. These observations suggest that aging and inflammation in the oral cavity evolve together, whereas the antimicrobial activity remains constant. Furthermore, the significant correlations observed among the eicosanoids and the marked association between LTB_4_ and MMP-9 activities in smokers would also suggest that the mediators of inflammation may originate from the same cellular source upon stimulation. Since these mediators are a cardinal sign of inflammation, the results indirectly suggest that the oral cavity of healthy subjects may be in a state of chronic inflammation. High levels of PGE_2_ have been correlated with periodontal disease [10] and were suggested to predict the evolution and severity of this disease [17]. One possible explanation for the results (present report) is that all healthy subjects without exception have periodontal disease and that elevated levels of PGE_2_ were a sign of this condition rather than age. However, that all healthy subjects have periodontal disease seems unlikely since Loe [18] elegantly demonstrated that gingival inflammation was not recognized in healthy subjects until at least 10 days following cessation of all measures of dental cleanliness and that 2–3 weeks without oral hygiene is necessary to produce mild inflammation. While no oral examination was performed on the healthy volunteers in the present investigation, all subjects performed basic oral hygienic measures. Unfortunately, there is little information available addressing gingival status in healthy subjects at different ages. Although lysozyme activity has not been correlated with either gingival inflammation [19] or age (this report), the interpretation that increased levels of PGE_2_ may be linked to periodontal disease and/or aging supports the notion that the oral cavity may be in a state of chronic low inflammation.

The detection of both cyclooxygenase and lipoxygenase metabolites in human saliva (present study) supports the data from previous investigations [5], [20], [21]. These mediators have been shown to be elevated in diseased subjects [10]. While the latter report indirectly suggested a relationship between inflammatory mediators in the oral cavity, the present report established a significant correlation between PGE_2_ and LTB_4_ in saliva from healthy subjects. The data, in addition, confirm and extend the observations of a preferential release of lipoxygenase metabolites in saliva from healthy subjects [7], [22] since LTB_4_ levels were predominantly detected compared with PGE_2_ at all ages. Increased levels of LTB_4_ have also been reported in gingival crevicular fluid from patients with periodontal disease and recent results have now confirmed these observations in the gingival crevicular fluid of patients with cardiovascular disease [11]. However, the present and previous [23] studies detecting nanogram levels of leukotrienes suggest that human whole buccal saliva contains approximately 5–10 fold more lipoxygenase products compared with the gingival crevicular fluid.

The high concentrations of LTB_4_ detected in human saliva are in contrast to the difficulty to detect this lipoxygenase product in blood and urine samples. The latter findings have been explained by the notion that LTB_4_ is thought only to be produced, released and act locally via cell membrane receptors at the site of the inflammatory response. Since the principal action of LTB_4_ is chemotaxis with a primary target being leukocytes, one would on this basis expect the oral cavity of healthy subjects to be overwhelmed with PNMs. However, this is not the case since healthy subjects are known to have only low levels of PMNs in the oral cavity [2], [3], [5]. Therefore, the role of LTB_4_ at nanogram concentrations in human saliva, which can be significantly reduced by lipoxygenase inhibitors [23], is presently difficult to explain. Nevertheless, a possible speculative explanation may be proposed based on the previous and the following observations. In the present study, the saliva was acidified to a pH of approximately 3 during analysis, confirming a resistance of leukotrienes to low pH [24] and suggesting a possible stability of salivary LTB_4_ during gastrointestinal (GI) passage. When saliva is swallowed (about 1.5 liters/24h) there may be sufficient quantities of LTB_4_ available to regulate the human GI-tract. This notion is further supported by the markedly lower ability of isolated human intestinal segments to convert arachidonic acid to leukotrienes compared with their production of PGE_2_ via local expression of the cyclooxygenase isozymes within the intestinal wall [25]. These observations suggest that salivary LTB_4_ may not only have local actions in the oral cavity, albeit, presently not known, but may also effect and regulate tissues in the GI-tract. The notion of a possible distant effect for the substantial quantities of LTB_4_ detected in the oral cavity is well within the potential for the capacity of salivary components to interact with the GI-tract as supported by the effects of smoking on inflammatory bowel disease [26], potential GI-tract mucosal protection by salivary derived nitric oxide in patients in intensive care units [27], [28] as well as the enhanced mediator release and altered ionic conditions in the esophageal mucosal barrier after stimulation of salivary secretion in patients with gastroesophageal reflux (GER) disease [29], [30]. Along similar lines, night-time GER is intimately linked to sleep apnea and hypertension [30] during a period when the swallowing rate and the production of saliva are markedly reduced. Finally, data from animal models have clearly linked salivary production of mediators with GI-tract regulation since sialoadenectomy markedly reduces luminal intestinal levels of epidermal growth factor in mice [31]. Although each of the complex conditions cited above may involve a variety of different mechanisms, the point to highlight is that components of saliva may be implicated in the regulation of the GI-tract. In view of the constant and considerable locally released quantities of LTB_4_ in the oral cavity of healthy human subjects, the concept of lipoxygenase metabolites acting only locally may have to be re-examined.

MMP-9 constitutes the dominant MMP activity in saliva [6], [9], [32], which was confirmed in the present report using similar gelatinolylic activity assays. However, in contrast to previous studies, MMP-9 activity was detected also in acidified saliva (present report). These observations support the notion that salivary MMP-9 activity may also be a potential mediator distal to gastric passage. Interestingly, mice with a targeted MMP-9 gene deletion display reduced extent and severity of bacterial induced enterocolitis [33]. In the present study, salivary MMP-9 activity was significantly higher in older subjects, hence reinforcing the notion of the oral cavity as a site of chronic inflammation associated with aging. Furthermore, in smokers, but not in non-smokers, MMP-9 activity was closely correlated to LTB_4_. Previous findings on the effects of smoking on leukotriene concentrations are contradictory with both neutral effects [11] and decreased [34] oral concentrations having been reported. While no alterations of LTB_4_ levels was observed in smokers compared with non-smokers in the present study, the alteration of the LTB_4_/MMP-9 correlation in smokers indicate interactions between these two pathways provoked by smoking.

In conclusion, the present study suggests that the inflammatory response as monitored by eicosanoid concentrations and MMP-9 activity in saliva is intimately linked with age, whereas the lysozyme antimicrobial activity may be independent. The presence of significant levels of these specific mediators of inflammation suggests that the oral cavity in man is in a constant low state of inflammation. While there are several mediators which act locally such as in periodontal disease, the observations cited above and the data (present report) suggest that there may be salivary factors acting as distant mediators in the GI-tract.

## Materials and Methods

### Subjects

This study group consisted of 94 healthy subjects (45 men and 49 women), whose ages ranged from 14 years to 76 years and had not taken aspirin in the week prior to the salivary sample. Included in these healthy volunteers was a group of 21 healthy smokers (less than 10 cigarettes/day). All subjects exercised basic measures of oral hygiene. Written and oral consent were obtained from each subject prior to the study. A written response from the national committee stated that this study did not require submission to the *Comité de Protection des Personnes*.

### Sample collection

Whole buccal saliva was collected from subjects within the first 30 min after waking in the morning. Subjects were either requested to collect 1 ml samples or individuals were requested to collect a sample during a 3 min period (16 men and 21 women). Samples were brought to the laboratory at the beginning of the work day. The saliva was diluted with de-ionized water (1∶4 v/v). The pH was adjusted to pH 3.5–4 with acetic acid and heated for 2 min at 100°C. The samples were then centrifuged (4000 rpm/10 min/4°C) and aliquots of 1 ml were stored at −20°C until analysis.

### Lysozyme assay

Lysozyme activity was determined spectrophotometrically by measuring the lysis of a suspension of *Micrococcus lysodeikticus* (3 mg/ml) [35]. Standard curves were constructed by incubation of egg white lysozyme (1 mg/ml) in 1.5 ml of 50 mM potassium phosphate buffer containing *M. lysodeikticus*, sodium azide (0.1%), BSA (1 mg/ml), mixed and incubated at 37°C for 2 h. An aliquot of 200 µl of each sample was added to 1.8 ml of potassium phosphate buffer containing the bacterial buffer as indicated. The pH of this working solution was between 7.1–7.4. Serial dilutions (nine) were performed and the changes in turbidity were monitored at a wavelength of 450 nm with potassium phosphate buffer as blank. A standard curve was plotted as optical density versus the concentration of lysozyme. The lysozyme activity detected in the fluids derived from the saliva were estimated from the standard curves and expressed as µg/ml. Flow rates in healthy volunteers (men: 0.68±0.09 µg/ml/min, n = 16 and women: 0.67±0.09 µg/ml/min, n = 21, were not significantly different). The lysozyme activities under flow controlled conditions were not different from data obtained from individuals who collected 1 ml of saliva. The mediator release and MMP-9 activities measured were also independent of flow.

### Eicosanoid assays

The levels of the different metabolites of arachidonic acid were determined using enzyme-immunoassays kits for prostaglandin E_2_ (PGE_2_) and leukotriene B_4_ (LTB_4_: Cayman Chemical Co USA). Measurements were made by following the instructions provided in the commercially available kits and were performed on the salivary fluids which had been diluted with water, adjusted to low pH and heated.

### MMP-9 gelatin zymographic assays

Gelatinolytic activities of pro-MMP-9 and MMP-9 were measured as previously described [36]. Briefly, the 20 µl of samples were mixed with loading buffer (50 mM TRIS–HCl, pH 6.8, 0.1% glycerol, 2% SDS, 0.5 mg/ml bromophenol blue). The electrophoresis was performed in 10 % polyacrylamide gels containing 2.5 mg/ml of gelatine (type IV) in TRIS-base-glycine buffer (0.124 M TRIS, 0.95 M glycine, 0.01% SDS) at 200V during 1 hour. Then, the gels were soaked two times for 30 min in 2.5% Triton X-100 at room temperature followed by brief rinse in distilled water. The incubation with 50 mM TRIS-HCl, pH 7.8, 10 mM CaCl_2 _buffer was performed for 19 h at 37°C. Gels were stained with Coomassie Brilliant Blue buffer (0.5% Coomassie Brilliant Blue R-250, 10% propanol, 5% acetic acid) on a shaker at room temperature, destained in 10% acetic acid, 30% ethanol (v/v) during 10 min and stored in 10% acetic acid solution (v/v). The inhibition profile of the gelatinolytic activities were performed by incubating the gels with 10 mM EDTA or 50 µg/ml Trasylol® (serine protease inhibitor).

### Calculations and Statistical analysis

All results are means±SEM. The pro-MMP-9 and MMP-9 activities were estimated from the two major bands obtained in the zymographic assays when judged to be in a linear range which corresponded to the gelatinolytic migration activity of a previously published standard [36]. This MMP-like activity was expressed in arbitrary units (AU). Statistical analysis for comparisons between age groups was performed by one-factor ANOVA, with post hoc comparisons made by Bonferroni-corrected paired tests. Linear associations were determined as Pearson's correlation coefficients (r). Multivariate analysis was also performed for investigating the associations between the measured parameters and a value of P<0.05 was taken as an indication of significance.
